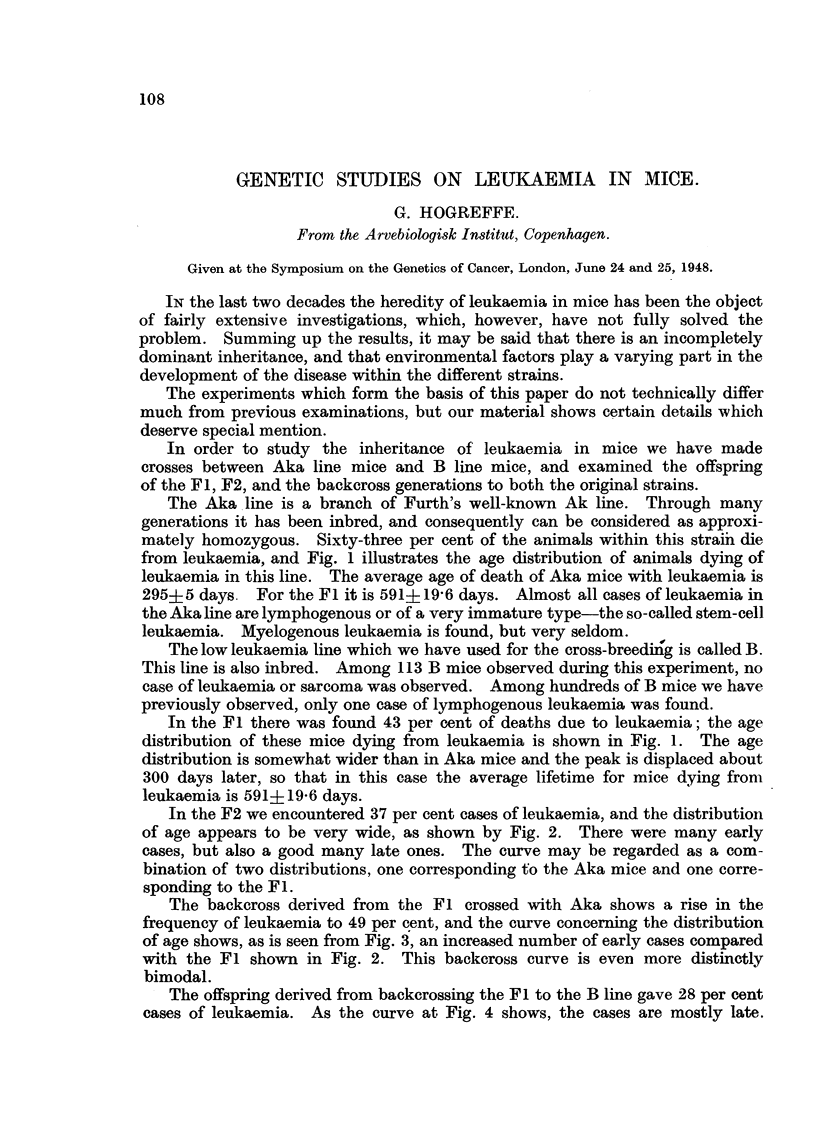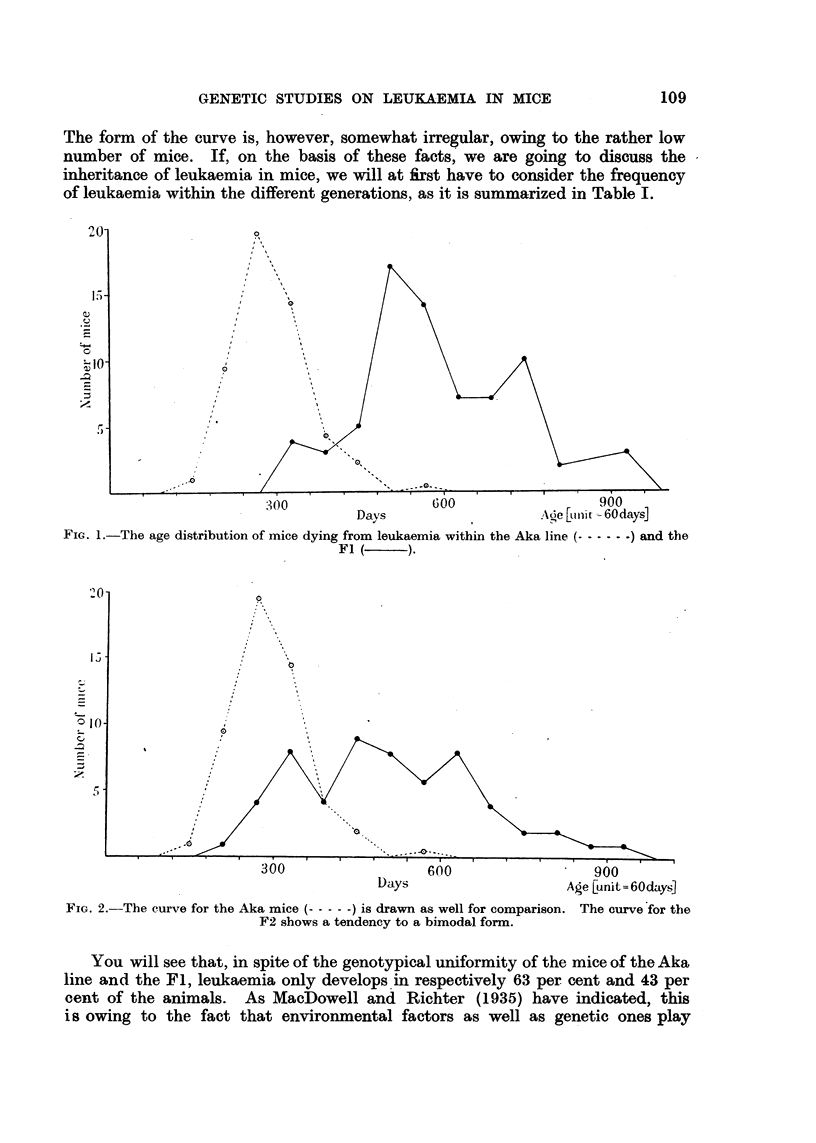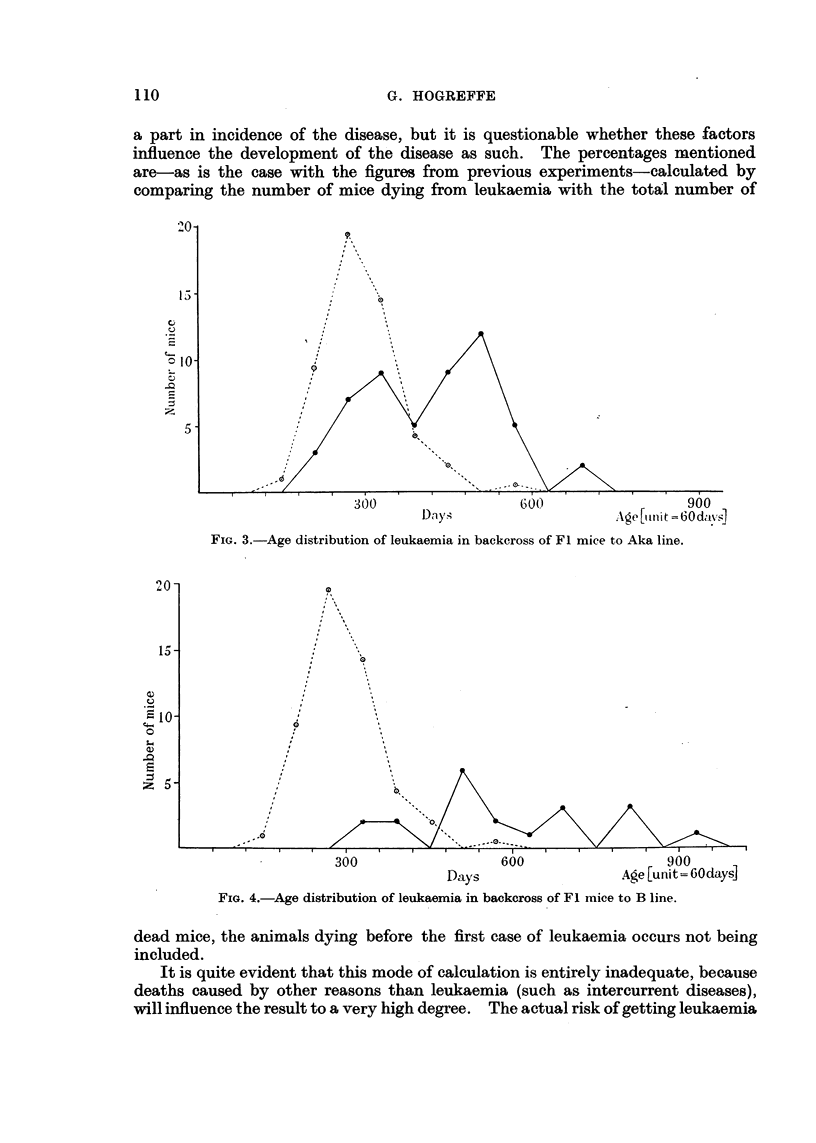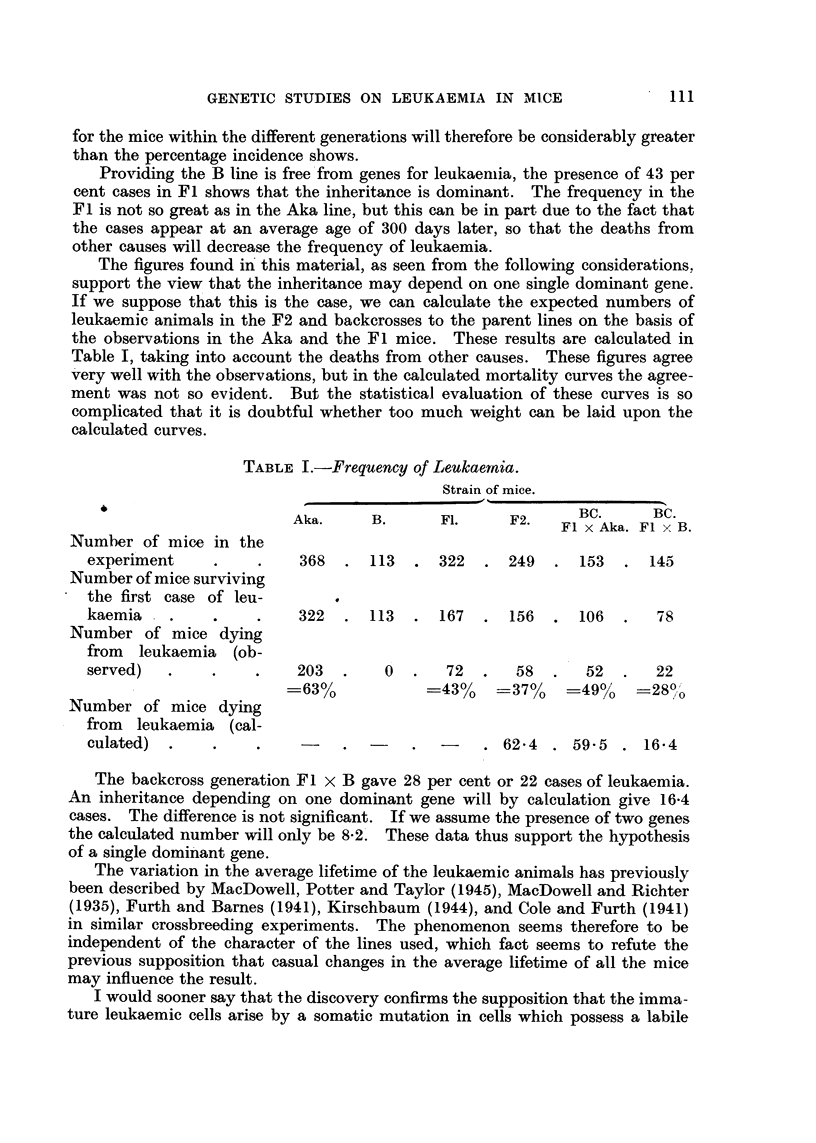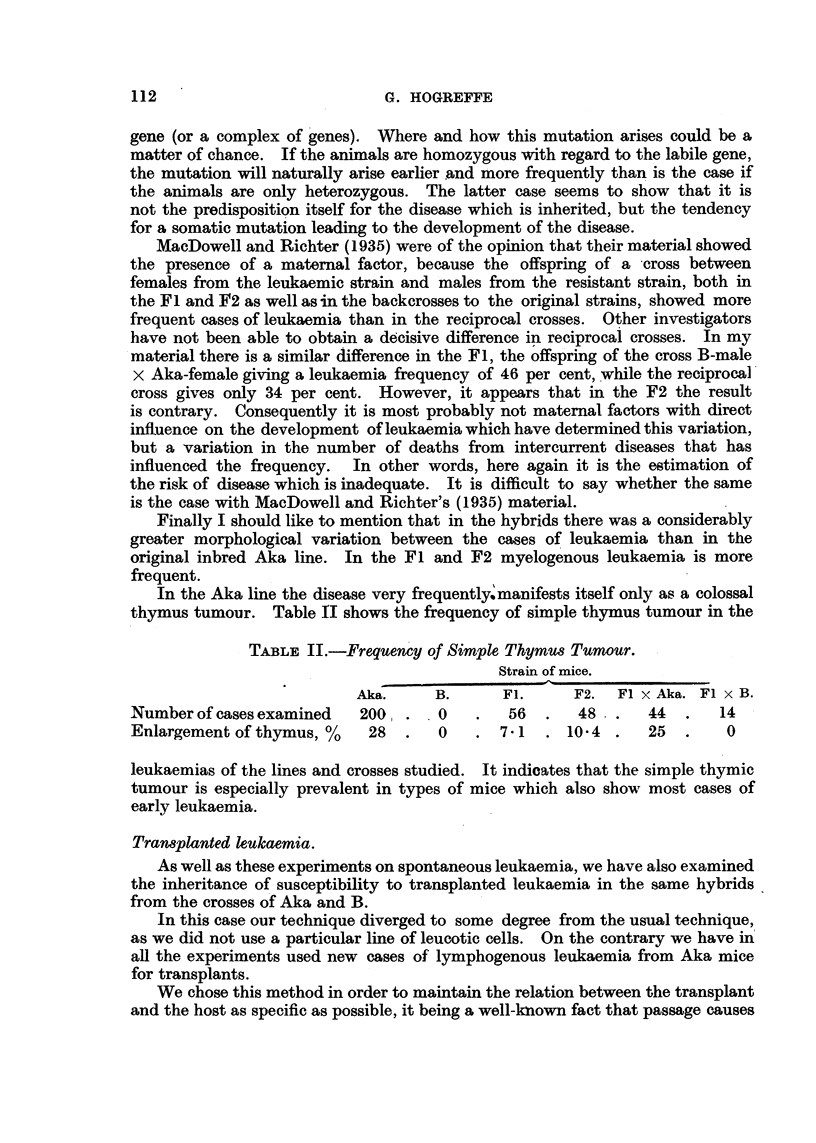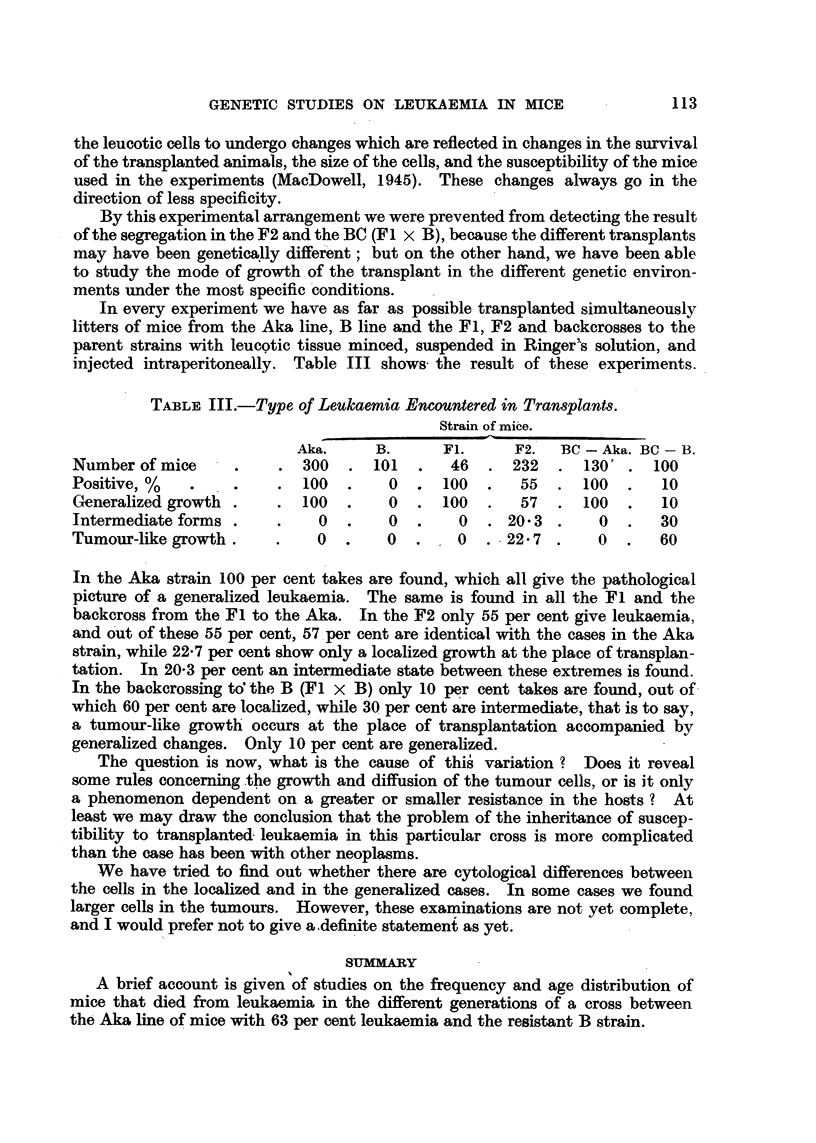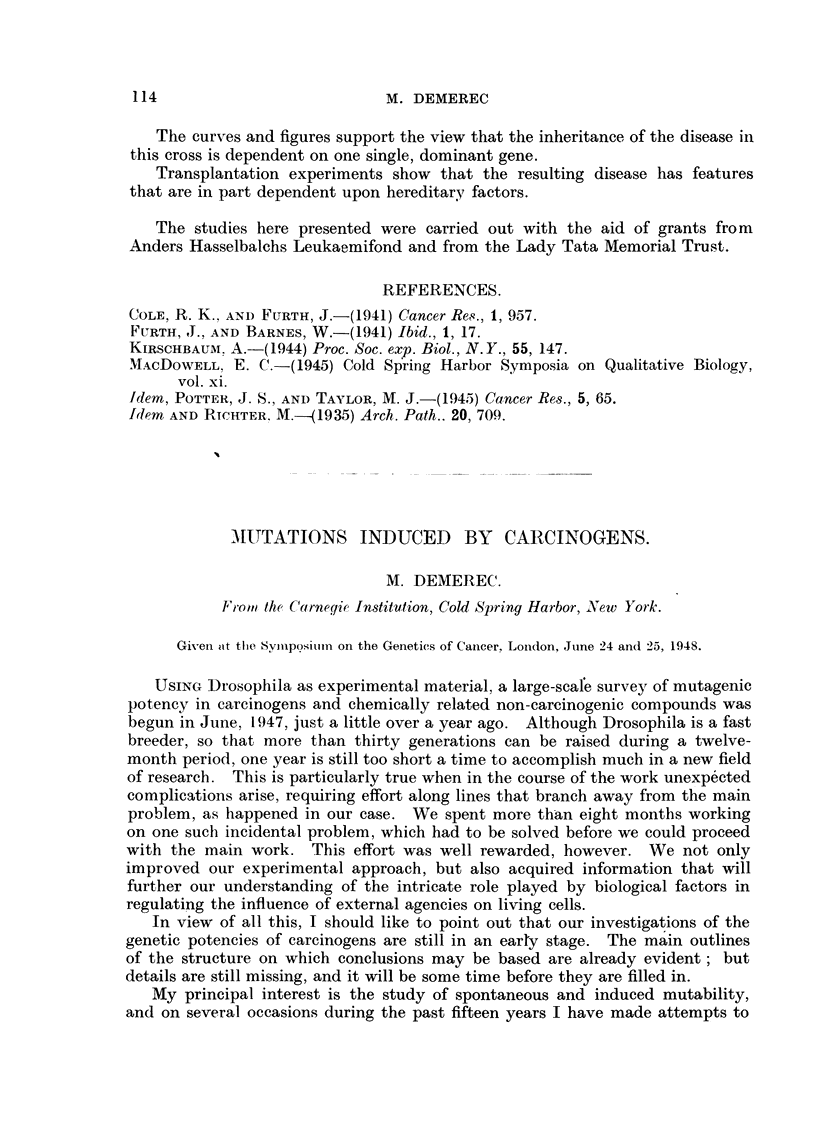# Genetic Studies on Leukaemia in Mice

**DOI:** 10.1038/bjc.1948.15

**Published:** 1948-06

**Authors:** G. Hogreffe


### GENETIC STUDIES ON LEUKAEMIA IN MICE.

G. HOGREFFE.

*From the Arvebiologisk Institut, Copenhagen.*

Given at the Symposium on the Genetics of Cancer, London, June 24 and 25, 1948.

In the last two decades the heredity of leukaemia in mice has been the object of fairly extensive investigations, which, however, have not fully solved the problem. Summing up the results, it may be said that there is an incompletely dominant inheritance, and that environmental factors play a varying part in the development of the disease within the different strains.

The experiments which form the basis of this paper do not technically differ much from previous examinations, but our material shows certain details which deserve special mention.

In order to study the inheritance of leukaemia in mice we have made crosses between Aka line mice and B line mice, and examined the offspring of the F1, F2, and the backcross generations to both the original strains.

The Aka line is a branch of Furth's well-known Ak line. Through many generations it has been inbred, and consequently can be considered as approximately homozygous. Sixty-three per cent of the animals within this strain die from leukaemia, and Fig. 1 illustrates the age distribution of animals dying of leukaemia in this line. The average age of death of Aka mice with leukaemia is $295 \pm 5$ days. For the F1 it is $591 \pm 19{\cdot}6$ days. Almost all cases of leukaemia in the Aka line are lymphogenous or of a very immature type—the so-called stem-cell leukaemia. Myelogenous leukaemia is found, but very seldom.

The low leukaemia line which we have used for the cross-breeding is called B. This line is also inbred. Among 113 B mice observed during this experiment, no case of leukaemia or sarcoma was observed. Among hundreds of B mice we have previously observed, only one case of lymphogenous leukaemia was found.

In the F1 there was found 43 per cent of deaths due to leukaemia; the age distribution of these mice dying from leukaemia is shown in Fig. 1. The age distribution is somewhat wider than in Aka mice and the peak is displaced about 300 days later, so that in this case the average lifetime for mice dying from leukaemia is $591 \pm 19{\cdot}6$ days.

In the F2 we encountered 37 per cent cases of leukaemia, and the distribution of age appears to be very wide, as shown by Fig. 2. There were many early cases, but also a good many late ones. The curve may be regarded as a combination of two distributions, one corresponding to the Aka mice and one corresponding to the F1.

The backcross derived from the F1 crossed with Aka shows a rise in the frequency of leukaemia to 49 per cent, and the curve concerning the distribution of age shows, as is seen from Fig. 3, an increased number of early cases compared with the F1 shown in Fig. 2. This backcross curve is even more distinctly bimodal.

The offspring derived from backcrossing the F1 to the B line gave 28 per cent cases of leukaemia. As the curve at Fig. 4 shows, the cases are mostly late.

The form of the curve is, however, somewhat irregular, owing to the rather low number of mice. If, on the basis of these facts, we are going to discuss the inheritance of leukaemia in mice, we will at first have to consider the frequency of leukaemia within the different generations, as it is summarized in Table I.

FIG. 1.—The age distribution of mice dying from leukaemia within the Aka line (- - - - - -) and the F1 (———).

FIG. 2.—The curve for the Aka mice (- - - - -) is drawn as well for comparison. The curve for the F2 shows a tendency to a bimodal form.

You will see that, in spite of the genotypical uniformity of the mice of the Aka line and the F1, leukaemia only develops in respectively 63 per cent and 43 per cent of the animals. As MacDowell and Richter (1935) have indicated, this is owing to the fact that environmental factors as well as genetic ones play a part in incidence of the disease, but it is questionable whether these factors influence the development of the disease as such. The percentages mentioned are—as is the case with the figures from previous experiments—calculated by comparing the number of mice dying from leukaemia with the total number of

FIG. 3.—Age distribution of leukaemia in backcross of F1 mice to Aka line.

FIG. 4.—Age distribution of leukaemia in backcross of F1 mice to B line.

dead mice, the animals dying before the first case of leukaemia occurs not being included.

It is quite evident that this mode of calculation is entirely inadequate, because deaths caused by other reasons than leukaemia (such as intercurrent diseases), will influence the result to a very high degree. The actual risk of getting leukaemia for the mice within the different generations will therefore be considerably greater than the percentage incidence shows.

Providing the B line is free from genes for leukaemia, the presence of 43 per cent cases in F1 shows that the inheritance is dominant. The frequency in the F1 is not so great as in the Aka line, but this can be in part due to the fact that the cases appear at an average age of 300 days later, so that the deaths from other causes will decrease the frequency of leukaemia.

The figures found in this material, as seen from the following considerations, support the view that the inheritance may depend on one single dominant gene. If we suppose that this is the case, we can calculate the expected numbers of leukaemic animals in the F2 and backcrosses to the parent lines on the basis of the observations in the Aka and the F1 mice. These results are calculated in Table I, taking into account the deaths from other causes. These figures agree very well with the observations, but in the calculated mortality curves the agreement was not so evident. But the statistical evaluation of these curves is so complicated that it is doubtful whether too much weight can be laid upon the calculated curves.

TABLE I.—*Frequency of Leukaemia.*

| | Strain of mice. | | | | | |
|---|---|---|---|---|---|---|
| | Aka. | B. | Fl. | F2. | BC. Fl × Aka. | BC. Fl × B. |
| Number of mice in the experiment | 368 | 113 | 322 | 249 | 153 | 145 |
| Number of mice surviving the first case of leukaemia | 322 | 113 | 167 | 156 | 106 | 78 |
| Number of mice dying from leukaemia (observed) | 203 =63% | 0 | 72 =43% | 58 =37% | 52 =49% | 22 =28% |
| Number of mice dying from leukaemia (calculated) | — | — | — | 62·4 | 59·5 | 16·4 |

The backcross generation F1 × B gave 28 per cent or 22 cases of leukaemia. An inheritance depending on one dominant gene will by calculation give 16·4 cases. The difference is not significant. If we assume the presence of two genes the calculated number will only be 8·2. These data thus support the hypothesis of a single dominant gene.

The variation in the average lifetime of the leukaemic animals has previously been described by MacDowell, Potter and Taylor (1945), MacDowell and Richter (1935), Furth and Barnes (1941), Kirschbaum (1944), and Cole and Furth (1941) in similar crossbreeding experiments. The phenomenon seems therefore to be independent of the character of the lines used, which fact seems to refute the previous supposition that casual changes in the average lifetime of all the mice may influence the result.

I would sooner say that the discovery confirms the supposition that the immature leukaemic cells arise by a somatic mutation in cells which possess a labile gene (or a complex of genes). Where and how this mutation arises could be a matter of chance. If the animals are homozygous with regard to the labile gene, the mutation will naturally arise earlier and more frequently than is the case if the animals are only heterozygous. The latter case seems to show that it is not the predisposition itself for the disease which is inherited, but the tendency for a somatic mutation leading to the development of the disease.

MacDowell and Richter (1935) were of the opinion that their material showed the presence of a maternal factor, because the offspring of a cross between females from the leukaemic strain and males from the resistant strain, both in the F1 and F2 as well as in the backcrosses to the original strains, showed more frequent cases of leukaemia than in the reciprocal crosses. Other investigators have not been able to obtain a decisive difference in reciprocal crosses. In my material there is a similar difference in the F1, the offspring of the cross B-male × Aka-female giving a leukaemia frequency of 46 per cent, while the reciprocal cross gives only 34 per cent. However, it appears that in the F2 the result is contrary. Consequently it is most probably not maternal factors with direct influence on the development of leukaemia which have determined this variation, but a variation in the number of deaths from intercurrent diseases that has influenced the frequency. In other words, here again it is the estimation of the risk of disease which is inadequate. It is difficult to say whether the same is the case with MacDowell and Richter's (1935) material.

Finally I should like to mention that in the hybrids there was a considerably greater morphological variation between the cases of leukaemia than in the original inbred Aka line. In the F1 and F2 myelogenous leukaemia is more frequent.

In the Aka line the disease very frequently manifests itself only as a colossal thymus tumour. Table II shows the frequency of simple thymus tumour in the leukaemias of the lines and crosses studied. It indicates that the simple thymic tumour is especially prevalent in types of mice which also show most cases of early leukaemia.

TABLE II.—*Frequency of Simple Thymus Tumour.*

| | Strain of mice. | | | | | |
|---|---|---|---|---|---|---|
| | Aka. | B. | F1. | F2. | F1 × Aka. | F1 × B. |
| Number of cases examined | 200 | 0 | 56 | 48 | 44 | 14 |
| Enlargement of thymus, % | 28 | 0 | 7·1 | 10·4 | 25 | 0 |

*Transplanted leukaemia.*

As well as these experiments on spontaneous leukaemia, we have also examined the inheritance of susceptibility to transplanted leukaemia in the same hybrids from the crosses of Aka and B.

In this case our technique diverged to some degree from the usual technique, as we did not use a particular line of leucotic cells. On the contrary we have in all the experiments used new cases of lymphogenous leukaemia from Aka mice for transplants.

We chose this method in order to maintain the relation between the transplant and the host as specific as possible, it being a well-known fact that passage causes the leucotic cells to undergo changes which are reflected in changes in the survival of the transplanted animals, the size of the cells, and the susceptibility of the mice used in the experiments (MacDowell, 1945). These changes always go in the direction of less specificity.

By this experimental arrangement we were prevented from detecting the result of the segregation in the F2 and the BC (F1 × B), because the different transplants may have been genetically different ; but on the other hand, we have been able to study the mode of growth of the transplant in the different genetic environments under the most specific conditions.

In every experiment we have as far as possible transplanted simultaneously litters of mice from the Aka line, B line and the F1, F2 and backcrosses to the parent strains with leucotic tissue minced, suspended in Ringer's solution, and injected intraperitoneally. Table III shows the result of these experiments.

TABLE III.—*Type of Leukaemia Encountered in Transplants.*

| | Strain of mice. | | | | | |
|---|---|---|---|---|---|---|
| | Aka. | B. | F1. | F2. | BC – Aka. | BC – B. |
| Number of mice | 300 | 101 | 46 | 232 | 130 | 100 |
| Positive, % | 100 | 0 | 100 | 55 | 100 | 10 |
| Generalized growth | 100 | 0 | 100 | 57 | 100 | 10 |
| Intermediate forms | 0 | 0 | 0 | 20·3 | 0 | 30 |
| Tumour-like growth | 0 | 0 | 0 | 22·7 | 0 | 60 |

In the Aka strain 100 per cent takes are found, which all give the pathological picture of a generalized leukaemia. The same is found in all the F1 and the backcross from the F1 to the Aka. In the F2 only 55 per cent give leukaemia, and out of these 55 per cent, 57 per cent are identical with the cases in the Aka strain, while 22·7 per cent show only a localized growth at the place of transplantation. In 20·3 per cent an intermediate state between these extremes is found. In the backcrossing to the B (F1 × B) only 10 per cent takes are found, out of which 60 per cent are localized, while 30 per cent are intermediate, that is to say, a tumour-like growth occurs at the place of transplantation accompanied by generalized changes. Only 10 per cent are generalized.

The question is now, what is the cause of this variation ? Does it reveal some rules concerning the growth and diffusion of the tumour cells, or is it only a phenomenon dependent on a greater or smaller resistance in the hosts ? At least we may draw the conclusion that the problem of the inheritance of susceptibility to transplanted leukaemia in this particular cross is more complicated than the case has been with other neoplasms.

We have tried to find out whether there are cytological differences between the cells in the localized and in the generalized cases. In some cases we found larger cells in the tumours. However, these examinations are not yet complete, and I would prefer not to give a definite statement as yet.

### SUMMARY

A brief account is given of studies on the frequency and age distribution of mice that died from leukaemia in the different generations of a cross between the Aka line of mice with 63 per cent leukaemia and the resistant B strain.

The curves and figures support the view that the inheritance of the disease in this cross is dependent on one single, dominant gene.

Transplantation experiments show that the resulting disease has features that are in part dependent upon hereditary factors.

The studies here presented were carried out with the aid of grants from Anders Hasselbalchs Leukaemifond and from the Lady Tata Memorial Trust.

### REFERENCES.

COLE, R. K., AND FURTH, J.—(1941) *Cancer Res.*, **1**, 957.
FURTH, J., AND BARNES, W.—(1941) *Ibid.*, **1**, 17.
KIRSCHBAUM, A.—(1944) *Proc. Soc. exp. Biol., N.Y.*, **55**, 147.
MACDOWELL, E. C.—(1945) Cold Spring Harbor Symposia on Qualitative Biology, vol. xi.
*Idem*, POTTER, J. S., AND TAYLOR, M. J.—(1945) *Cancer Res.*, **5**, 65.
*Idem* AND RICHTER, M.—(1935) *Arch. Path.*, **20**, 709.

### MUTATIONS INDUCED BY CARCINOGENS.

M. DEMEREC.